# Resource-Adaptive Semantic Transmission and Client Scheduling for OFDM-Based V2X Communications

**DOI:** 10.3390/s26092615

**Published:** 2026-04-23

**Authors:** Jiahao Liu, Yuanle Chen, Wei Wu, Feng Tian

**Affiliations:** 1 College of Telecommunications and Information Engineering, Nanjing University of Posts and Telecommunications, Nanjing 210023, China; b23012013@njupt.edu.cn (J.L.); 1225013528@njupt.edu.cn (Y.C.); 2 Key Laboratory of Broadband Wireless Communication and Sensor Network Technology, Ministry of Education, Nanjing University of Posts and Telecommunications, Nanjing 210023, China; tianf@njupt.edu.cn

**Keywords:** semantic communication, OFDM resource adaptation, adaptive transmission, client scheduling, vehicle-to-everything (V2X), channel-aware resource allocation, integrated sensing and communication

## Abstract

Proportional, fair scheduling in OFDM-based vehicle-to-everything (V2X) uplink causes the resource-block allocation of each vehicle to vary from slot to slot, yet conventional semantic encoders produce a fixed number of output tokens regardless of the instantaneous channel capacity. When the encoder output exceeds the slot budget, transmitted features are truncated and the resulting federated learning gradient is corrupted—a problem that affected 23% of training rounds for non-line-of-sight vehicles in our experiments. The difficulty is worsened by a spatial pattern common in urban deployments: vehicles at congested intersections suffer the poorest propagation conditions while carrying the training data most relevant to safety, and throughput-driven client selection excludes them in favor of vehicles with strong channels but uninformative scenes. We address both issues within a single framework for OFDM-based V2X federated learning. On the transmission side, a Sensing-Guided Adaptive Modulation (SGAM) module derives a per-slot token budget from the current resource-block allocation and selects tokens through differentiable Gumbel-TopK pruning with a hard capacity clip, so the transmitted token count stays within the slot budget. On the scheduling side, a Channel-Decoupled Federated Learning (CDFL) module partitions clients independently by channel quality and data complexity, selects diverse representatives per partition via facility location optimization, and corrects for partition-size imbalance through inverse propensity weighting during model aggregation. Experiments on NuScenes with 20 non-IID vehicular clients under realistic OFDM channel simulation demonstrate a Macro-F1 of 0.710 (+8.7 points over the Oort-adapted baseline), zero budget violations throughout training, and a 75% reduction in training variance; the worst-class F1 more than doubles relative to FedAvg.

## 1. Introduction

### 1.1. Problem and Motivation

**Figure 1:**
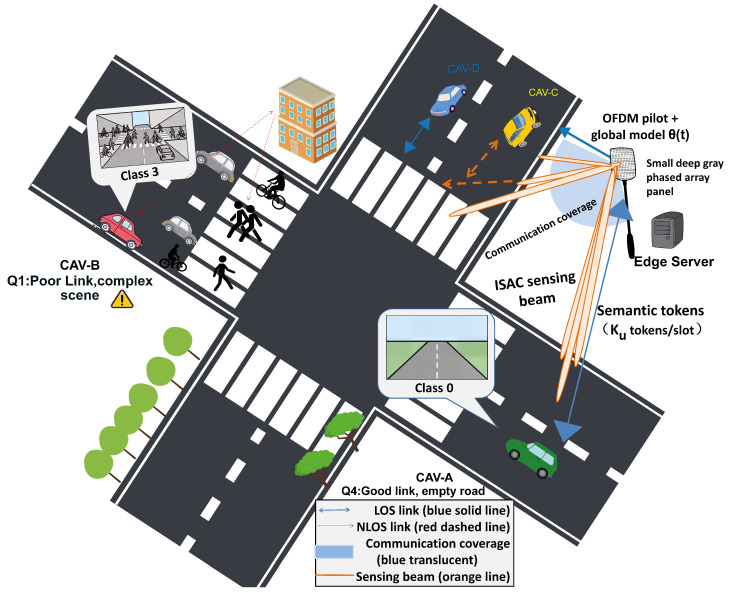
V2X cooperative perception scenario at a signalized intersection. The RSU transmits dual-function OFDM waveforms for downlink sensing and uplink data collection. Vehicles (CAVs) at different positions experience correlated disparities in both channel quality (path loss, LOS/NLOS) and scene complexity (object density, occlusion). Solid lines denote LOS communication links; dashed lines denote NLOS links; orange lines denote sensing beams.

Cooperative perception in vehicle-to-everything (V2X) networks relies on Connected and Automated Vehicles (CAVs) uploading processed sensor features to an edge server through an OFDM uplink. As illustrated in Figure 1, at a signalized intersection the RSU transmits dual-function OFDM waveforms for downlink sensing and uplink data collection, while vehicles at different positions experience correlated disparities in both channel quality and scene complexity. Semantic communication makes this feasible at limited spectrum cost by transmitting only compact, task-relevant tokens rather than raw sensor data [1,2]. The difficulty is that the encoder output must fit a channel whose capacity is not fixed.

Under proportional fair scheduling with frequency-selective fading, the resource-block set assigned to each vehicle is recomputed every transmission time interval based on instantaneous channel state information (CSI), competing traffic load, and quality-of-service priorities. A vehicle that receives 40 subcarriers in one slot may be allocated only 12 in the next. In our 20-vehicle OFDM simulation the per-slot token capacity for a single vehicle ranged from 8 to 49 across federated learning (FL) rounds. An encoder with a fixed output of 32 tokens cannot track this variation: when the allocation drops below 32, the physical layer discards the excess and the uplink transmission carries an incomplete feature set. Among non-line-of-sight (NLOS) vehicles in our setup, 23% of training rounds suffered such overflow events, wasting both the spectrum occupied by the discarded symbols and the on-board computation spent producing them.

The overflow does not affect all vehicles equally. Urban V2X deployments exhibit a persistent spatial pattern: vehicles sitting behind buildings at crowded intersections face deep fading, strong multipath, and high Doppler spread, while simultaneously recording the densest and most occluded driving scenes. These are exactly the scenes that demand cooperative perception, yet a scheduling rule that ranks vehicles by the product of training loss and uplink throughput—following the logic of Oort [3]—penalizes them for providing a poor channel. In our experiments such vehicles were selected for FL participation in only 8% of rounds, compared with 42% for vehicles on open roads with simple scenes and strong line-of-sight. Over many rounds, the global model drifted toward the easy-case data that dominates the selected pool and performed poorly on the complex intersection scenarios that matter most for safety.

Prior work treats the resource-adaptation problem and the client-scheduling problem in isolation. Deep joint source–channel coding (JSCC) methods adapt the encoder to the channel but typically assume a slowly varying or fixed allocation within each transmission interval [4,5]. Heterogeneous FL methods such as FedProx [6] and SCAFFOLD [7] mitigate gradient drift under non-IID data, but treat the communication link purely as a latency or throughput cost, without accounting for its correlation with the data distribution. The joint design of per-slot semantic rate control and channel-aware client scheduling has not been attempted in vehicular OFDM networks.

### 1.2. Contributions

We propose a framework that coordinates semantic transmission rate and client scheduling within the OFDM resource structure:

- A Sensing-Guided Adaptive Modulation (SGAM) module that maps each slot’s resource-block allocation to a hard token budget, conditions the backbone features on ISAC-derived physical-layer parameters (range, velocity, sensing confidence) through Feature-wise Linear Modulation (FiLM), and enforces the budget via Gumbel-Top*K* selection with a capacity clip. The capacity clip prevents budget violations, and the average uplink payload drops by 38% at equal task performance.
- A Channel-Decoupled Federated Learning (CDFL) module that independently partitions clients by channel quality and data complexity into four quadrants, selects diverse representatives per quadrant through facility location optimization, and applies inverse propensity weighting during model aggregation to correct for unequal quadrant sizes. The selection rate of the most underserved vehicle group rises from 8% to 22%.
- Experimental validation on NuScenes with 20 non-IID vehicular clients under OFDM-level channel simulation with Rayleigh/Rician fading and proportional fair scheduling. The framework achieves 0.710 Macro-F1 (+8.7 points over the Oort-adapted baseline), zero budget violations, 75% lower training variance, and worst-class F1 more than doubled relative to FedAvg. We also provide an $O(1/\sqrt{T})$ convergence bound under standard non-convex assumptions.

Beyond the ground-based RSU deployment evaluated in our experiments, the proposed framework extends naturally to UAV-assisted V2X networks, in which a UAV serves as an aerial RSU to provide coverage at congested urban intersections. In such deployments the air-to-ground channel exhibits altitude-dependent Rician *K*-factor variability and stronger slot-level resource fluctuations than terrestrial links [8], making the adaptive token budgeting of SGAM particularly valuable. The limited on-board energy of a UAV further amplifies the benefit of the 38% bandwidth reduction achieved by SGAM. The channel–data coupling addressed by CDFL persists in UAV-V2X: vehicles beneath the UAV enjoy strong links but may observe simple scenes, while vehicles at the coverage boundary face degraded channels with complex occluded views. We discuss this extension further in Section 9.

The rest of the paper is structured as follows. Section 2 surveys related work on semantic communication, heterogeneous federated learning, and integrated sensing and communication. Section 3 defines the system model and formulates the optimization problem. Section 4 and Section 5 detail the SGAM and CDFL modules. Section 6 describes the training objective. Section 7 provides a convergence bound. Section 8 reports experimental results. Section 9 discusses limitations, and Section 10 concludes. Finally, Appendix A provides the complete algorithmic pseudocode for the training procedure, and Appendix B summarizes the key mathematical symbols used throughout the paper.

## 2. Related Work

### 2.1. Semantic Communication for V2X

Rather than reconstructing the source signal at the bit level, semantic communication transmits task-relevant information for the downstream objective [1,2]. Deep JSCC is the most studied realization: an encoder–decoder pair trained end-to-end maps source data directly to channel symbols, bypassing the separation principle. Across image, text, and speech modalities, learned JSCC consistently outperforms separate source and channel coding at low SNR [4,5]. In vehicular networks, reinforcement-learning-based methods dynamically adjust encoding granularity and spectrum allocation under time-varying channels [9,10], and 6G-oriented semantic image transmission designs have reported further bandwidth savings [11].

A recurring limitation across these methods is that the encoder is either trained for a fixed channel or conditioned on a single slowly varying channel realization per transmission interval [4]. In vehicular OFDM the proportional fair scheduler reassigns resource blocks to every slot on the basis of instantaneous CSI and competing load. A vehicle allocated 40 subcarriers in one slot may receive only 12 in the next; retraining the encoder at millisecond timescales is not feasible. Variable-rate learned compression [12] can produce outputs of different lengths but has not been integrated with OFDM resource-block constraints or federated training procedures.

In the D2D vehicular context, Su et al. [13] proposed a cross-layer resource allocation framework that combines semantic access control at the application layer with robust power control at the physical layer under imperfect CSI. Their formulation uses Lyapunov optimization to decompose a long-term problem into per-slot subproblems and employs Bernstein approximation to handle chance constraints arising from vehicle mobility. This work demonstrates the value of coupling semantic transmission decisions with physical-layer resource management—a principle shared by our SGAM module. However, their framework operates in a centralized single-cell D2D setting with a fixed semantic encoder output size and does not address per-slot variation in the number of OFDM resource blocks available to each vehicle. Furthermore, the D2D pair model does not involve federated training across multiple heterogeneous vehicles, where the correlation between channel quality and local data complexity creates a client selection bias that our CDFL module is designed to resolve.

### 2.2. Heterogeneous Federated Learning

When local data distributions diverge substantially, FL convergence slows and the global model may oscillate between conflicting optima [6,7,14]. FedProx [6] limits local drift with a proximal penalty $\frac{\mu }{2}{∥w-w^{t}∥}^{2}$. SCAFFOLD [7] corrects gradient bias through per-client control variates but doubles the per-round communication cost. For client selection, Oort [3] scores each client by the product of training loss and inverse completion time, favoring informative data on fast links, and TiFL [15] groups clients into latency tiers and samples from each tier. Neither method accounts for the possibility that channel quality and local data distribution are correlated.

In vehicular FL this assumption fails. A vehicle in an urban canyon with deep fading is likely observing a complex scene with dense traffic and heavy occlusion. Utility-based selection penalizes such vehicles twice—high training loss offset by low throughput—and over many rounds the selected pool becomes dominated by vehicles with favorable channels and simple data [16,17]. Clustered FL approaches [18] group clients by gradient similarity but do not exploit channel-side information for the grouping.

The channel–data coupling bias exposed by utility-based FL selection is a manifestation of the same physical-layer dynamics that ISAC (Section 2.3) can potentially resolve through sensing-derived context.

### 2.3. Integrated Sensing and Communication

ISAC shares the OFDM waveform between radar sensing and data communication, using spectrum and hardware jointly [19,20]. Waveform design seeks to balance sensing accuracy—bounded by the Cramér–Rao bound (CRB)—against communication throughput [21,22]. Delay–Doppler processing allows sensing-derived target parameters to assist channel estimation, reducing pilot overhead [23]. Object-oriented ISAC formulations organize per-target state vectors for communication system design [24]. A comprehensive survey identified vehicular networks as a prime ISAC domain and noted that application-layer exploitation of sensing information remains largely unexplored [25].

In this work the RSU’s sensing estimates (range, velocity, angle, and a derived confidence score) serve as side information for the semantic encoder through FiLM conditioning [26]. The ablation study in Section 8 shows that this integration improves worst-class F1 from 0.410 to 0.465, with the benefit concentrated on high-difficulty scenes where backbone features alone are insufficient. A tighter form of integration—for instance, spatially weighting the feature map using CRB-derived location estimates—remains a direction for future investigation.

### 2.4. Summary and Research Gap

The three research streams surveyed above have each advanced substantially in isolation. However, their intersection in vehicular OFDM networks exposes a gap that existing methods do not address. Semantic communication methods [1,2,4,5,9,10] optimize encoder–decoder pairs for task-relevant transmission but assume either a fixed channel allocation or a slowly varying SNR within each transmission interval. Classical variable-rate compression [12] adapts the bottleneck bitrate for a continuous rate-distortion trade-off, yet none of these approaches account for the slot-level resource-block volatility imposed by proportional fair scheduling in multi-user OFDM, where a vehicle’s subcarrier count can change by a factor of four between consecutive slots. Heterogeneous FL methods [3,6,7,15] mitigate gradient drift under non-IID data distributions but treat the communication channel as a latency or throughput cost independent of the local data distribution. In V2X networks this independence assumption breaks down: vehicles at congested intersections simultaneously experience poor propagation and observe the most complex driving scenes, creating a systematic correlation between channel quality and data utility that throughput-driven schedulers exploit adversely. ISAC waveform designs [19,20,21,22,23,24] demonstrate that sensing-derived environmental context can improve physical-layer operations, yet the application-layer potential of this context remains largely unexploited. The joint problem of per-slot semantic rate adaptation and channel-aware client scheduling under federated training in vehicular OFDM networks has not been addressed. Table 1 positions representative prior works against three capability dimensions to highlight this gap.

## 3. System Model and Problem Formulation

**Figure 2:**
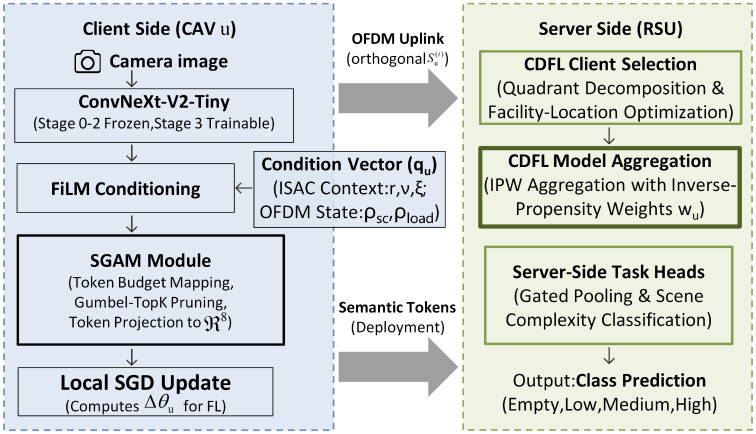
End-to-end architecture of SIFL-V2X. **Left**: SGAM on the client side. **Right**: CDFL on the server side.

We consider a cellular V2X network comprising a single roadside unit (RSU) with an edge server and a set U={1,…,U} of vehicular clients. The system operates over *T* FL rounds. Figure 2 shows the end-to-end architecture.

### 3.1. ISAC-OFDM Physical Layer

The system uses an OFDM waveform with $N_{\mathrm{sc}}=128$ subcarriers and spacing Δf for both uplink communication and downlink sensing. This configuration follows the 3GPP NR sidelink numerology specified in TS 38.211 [27] for a 10 MHz channel bandwidth with 15 kHz subcarrier spacing, representing a partial bandwidth allocation typical of V2X Mode 2 sidelink operation. The framework is not restricted to this specific value: the token budget $K_{\max}^{\left(t\right)}$ in Equation (7) adapts automatically to any $N_{\mathrm{sc}}$ through the slot capacity $B_{\mathrm{slot}}$.

#### 3.1.1. Communication Channel

The received signal at the RSU from client *u* on subcarrier *k* at round *t* is

(1) $$y_{u,k}=\sqrt{P_{u,k}}\;h_{u,k}^{\left(t\right)}\;s_{u,k}+n_{u,k},$$

where $h_{u,k}^{\left(t\right)}\in C$ combines path loss, log-normal shadowing, and small-scale fading; $P_{u,k}$ is the transmit power on subcarrier *k*; $s_{u,k}$ is the unit-energy data symbol ($E[|s_{u,k}{|}^{2}]=1$); and $n_{u,k}∼\mathrm{CN}\left(0,\sigma^{2}\right)$ is AWGN. The RSU runs a proportional fair scheduler that assigns each vehicle an orthogonal subcarrier set $S_{u}^{\left(t\right)}\subset \left\{1,\ldots ,N_{\mathrm{sc}}\right\}$, with $S_{u}\cap S_{v}=\emptyset$ for u≠v.

Small-scale fading follows a Rician model whose *K*-factor ranges from 0 dB (linear K=1, corresponding to equal LOS/NLOS power) to 10 dB (linear K=10, strong LOS). We note that 0 dB does *not* correspond to Rayleigh fading; the Rayleigh limit requires K→0 in linear scale (−∞ dB), in which no specular component exists. Our simulation therefore covers regimes from equal-power LOS/NLOS to strongly LOS-dominated propagation, representative of urban V2X deployments where a residual specular path persists even in partially obstructed geometries. The per-client instantaneous SNR spans [5,25] dB, representing propagation conditions from deep urban canyons to open suburban roads under varying path loss and shadowing.

#### 3.1.2. Sensing Model

Downlink pilots enable mono-static sensing at the RSU. The reflected echo from each vehicle yields an estimate of the parameter vector $p_{u}={\left[r_{u},v_{u},\theta_{u}\right]}^{⊤}$ (range, radial velocity, angle of arrival). For an OFDM-based ISAC waveform in a single-target scenario, the diagonal elements of the Fisher information matrix (FIM) $J_{u}$ are [28]

(2) $$\begin{array}{cc} {\left[J\right]}_{rr} & =\frac{8\pi^{2}B^{2}\gamma }{c^{2}}, \end{array}$$

(3) $$\begin{array}{cc} {\left[J\right]}_{vv} & =\frac{8\pi^{2}T_{d}^{2}f_{c}^{2}\gamma }{c^{2}}, \end{array}$$

where *B* is the effective signal bandwidth, $T_{d}$ is the coherent dwell time, $f_{c}$ is the carrier frequency, *c* is the speed of light, and γ is the received sensing SNR. These expressions follow from the standard Fisher information analysis for joint delay–Doppler estimation in OFDM radar waveforms [28]. The CRB for the full parameter vector is

(4) $$\mathrm{CRB}\left(p_{u}\right)=\mathrm{tr}\left(J_{u}^{-1}\right).$$

We compress this into a scalar sensing confidence $\xi_{u}\in \left[0,1\right]$:

(5) $$\xi_{u}=\frac{1}{1+\beta \cdot \mathrm{tr}(\mathrm{CRB}\left(p_{u}\right))},$$

where β is a normalization constant. Values near 1 correspond to precise parameter estimates; values near 0 indicate unreliable estimates due to low sensing SNR or short dwell time. This scalar enters the semantic encoder as part of the condition vector defined in Section 4.

### 3.2. Semantic Transmission and FL Formulation

Each client *u* holds a local dataset $D_{u}={\left\{\left(x_{u,i},y_{u,i}\right)\right\}}_{i=1}^{D_{u}}$ of camera images labeled with scene complexity classes 0–3. The labels are obtained by quantile-binning per-frame object density and occlusion ratio into four balanced categories: empty (Class 0), low complexity (Class 1), medium complexity (Class 2), and high complexity (Class 3).

The model consists of a semantic encoder $f_{\theta }\left(\cdot \right)$ that resides on the vehicle and maps raw images to compressed features, and task heads $g_{\phi }\left(\cdot \right)$ at the server that produce predictions from the received features. During FL training each vehicle holds a local copy of the full model and computes losses locally; at deployment time only the encoder runs on the vehicle and the selected tokens are transmitted over the OFDM uplink.

#### 3.2.1. Transmission Budget Constraint

**Figure 3:**
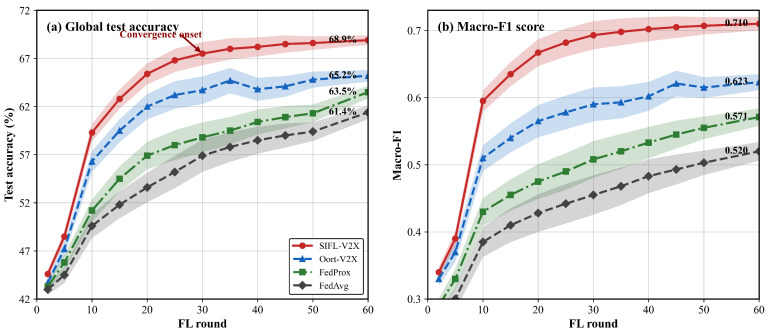
Test accuracy and Macro-F1 over 60 FL rounds. Shaded bands show ±1 standard deviation over three runs.

The number of usable bits per OFDM slot is determined by the allocated subcarriers, the modulation and coding scheme, and protocol overheads:

(6) $$B_{\mathrm{slot}}=\left|S\right|\cdot N_{\mathrm{sym}}\cdot \log_{2}\left(M\right)\cdot R_{c}\cdot \left(1-\rho_{\mathrm{pilot}}\right)\cdot \left(1-\rho_{\mathrm{cp}}\right),$$

where *M* is the modulation order, $R_{c}$ is the coding rate, $N_{\mathrm{sym}}$ is the number of OFDM symbols per slot, and $\rho_{\mathrm{pilot}}$, $\rho_{\mathrm{cp}}$ are the fractional overheads for pilot symbols and cyclic prefix. Each semantic token is compressed to a bottleneck dimension $d_{b}$ and quantized to *q* bits, giving $b_{\mathrm{token}}=d_{b}\times q$ bits per token. The maximum token count that fits a single slot is

(7) $$K_{\max}^{\left(t\right)}=\mathrm{clip}\;\left(\left\lfloor \frac{B_{\mathrm{slot}}}{b_{\mathrm{token}}}\right\rfloor ,\;1,\;L\right),$$

where L=49 is the total number of patch tokens produced by the backbone. The lower clip of 1 guarantees at least one token is transmitted even under severely degraded channels. Because channel fading causes |S| to fluctuate across rounds, $K_{\max}^{\left(t\right)}$ varies per client per round. A fixed-rate encoder whose output exceeds $K_{\max}^{\left(t\right)}$ loses the surplus to truncation, and the gradient computed from the incomplete features is no longer a reliable estimate. The severity of this resource volatility is illustrated by the convergence trajectories in Figure 3 (Section 8), where fixed-*K* baselines exhibit wide confidence bands that the proposed adaptive method eliminates. The choice of $K_{\mathrm{fixed}}=32$ (the median of the observed $K_{\max}$ range) represents a practical operating point that balances capacity utilization against violation risk; any fixed choice faces the same dilemma—lower values waste the capacity of good-channel slots and higher values increase violations in poor-channel slots—which motivates this adaptive approach.

#### 3.2.2. Optimization Objective

The global FL objective minimizes the weighted empirical risk across all clients:

(8) $$\min_{\theta ,\phi }\;L\left(\theta ,\phi \right)=\sum_{u=1}^{U}\frac{D_{u}}{D_{\mathrm{total}}}E_{\left(x,y\right)∼D_{u}}\left[\ell \left(g_{\phi }\left(f_{\theta }\left(x\right)\right),y\right)\right],$$

where $D_{u}=\left|D_{u}\right|$ is the number of samples held by client *u*, $D_{\mathrm{total}}=\sum_{u=1}^{U}D_{u}$ is the aggregate dataset size, ℓ(·,·) denotes the per-sample loss function (defined in Section 6), and $f_{\theta }$, $g_{\phi }$ are the semantic encoder and task head parameterized by θ and ϕ, respectively. This objective is subject to $K_{u}\leq K_{\max}^{\left(t\right)}$ for every selected vehicle in every round. Scene complexity classification serves as a semantic gate for the cooperative perception pipeline: a Class 0 prediction (empty road) triggers only a lightweight status message consuming negligible uplink spectrum, while a Class 3 prediction (complex intersection) triggers full cooperative feature sharing that requires substantial resource-block allocation. The classification accuracy of this gate directly determines how efficiently the system distributes its limited uplink capacity across the vehicle fleet.

## 4. SGAM: Adaptive Semantic Token Scheduling

SGAM sits between the backbone encoder and the OFDM transmission layer. Its role is to match the number of transmitted tokens to the resource budget of the current slot. The module operates in two stages: it first conditions the feature representation on the current physical-layer state through FiLM modulation, then determines a token count from the resource-block allocation and selects exactly that many tokens through Gumbel-Top*K* pruning. The logic mirrors conventional adaptive modulation and coding (AMC), which switches modulation order and coding rate per slot to match channel quality; SGAM applies the same principle one layer up by switching the semantic compression rate instead of the modulation order.

### 4.1. Channel-Conditioned Feature Extraction

A ConvNeXt-V2-Tiny backbone pretrained on ImageNet-22k produces a feature map $Z_{\mathrm{base}}\in R^{L\times d}$ with L=49 patch tokens and d=768 channels. Stages 0–2 are frozen; only stage 3 is updated during federated training.

The encoder’s internal representation is conditioned on the current channel and mobility state through a six-dimensional vector $q_{u}\in {\left[0,1\right]}^{6}$:

(9) $$q_{u}=\left[\frac{r_{u}}{r_{\max}},\;\frac{v_{u}}{v_{\max}},\;\frac{{\mathrm{SNR}}_{u}}{{\mathrm{SNR}}_{\max}},\;\xi_{u},\;\rho_{\mathrm{sc}},\;\rho_{\mathrm{load}}\right],$$

where $r_{u}$ and $v_{u}$ are the ISAC-derived range and radial velocity, $\xi_{u}$ is the sensing confidence defined in Equation (5), $\rho_{\mathrm{sc}}=\left|S_{u}\right|/N_{\mathrm{sc}}$ is the fraction of subcarriers allocated to vehicle *u*, and $\rho_{\mathrm{load}}$ is the ratio of concurrently active users to the maximum supported count. Normalization constants are $r_{\max}=400$ m, $v_{\max}=50$ m/s, and ${\mathrm{SNR}}_{\max}=30$ dB.

A two-layer MLP with architecture [6,256,1536] and GELU activation maps $q_{u}$ to per-channel scale and shift parameters for FiLM [26]:

(10) $${\tilde{z}}_{l}=\gamma \left(q_{u}\right)⊙z_{\mathrm{base},l}+\beta \left(q_{u}\right),\;l=1,\ldots ,L,$$

where $\left[\gamma ,\beta \right]={\mathrm{MLP}}_{\mathrm{FiLM}}\left(q_{u}\right)$ with $\gamma ,\beta \in R^{d}$, and ⊙ denotes element-wise multiplication. The learned γ tends to attenuate feature channels that are degraded under the current propagation conditions—texture channels corrupted by Doppler blur at high speed, for example—while the additive shift β compensates by encouraging the encoder to rely more heavily on more robust structural channels. At low speeds with strong line-of-sight, the modulation collapses toward identity mapping and the backbone output passes through largely unchanged. The ablation in Section 8 confirms that removing FiLM has limited impact on overall accuracy (−3.1 points) but drops worst-class F1 sharply from 0.465 to 0.310, indicating that conditioning is most valuable for difficult scenes under adverse channel and mobility conditions.

### 4.2. Token Budget and Selection

The token budget $K_{u}$ for vehicle *u* in round *t* derives from the physical-layer allocation. Starting from the instantaneous capacity $K_{\max}^{\left(t\right)}$ computed in Equation (7), a lightweight MLP produces a learned adjustment:

(11) $$K_{u}=\mathrm{clip}\;\left(K_{\max}^{\left(t\right)}+\Delta K\left(q_{u}\right),\;K_{\min},\;K_{\max}^{\left(t\right)}\right),$$

with $K_{\min}=4$. This formulation has two useful properties. First, the upper clip equals $K_{\max}^{\left(t\right)}$, so $K_{u}$ can never exceed the physical budget regardless of what the MLP outputs. Second, ΔK can only *reduce* the token count below $K_{\max}^{\left(t\right)}$ when the scene content is simple enough that fewer tokens suffice—an empty highway, for instance, does not benefit from all 49 tokens even when the channel could carry them. In such cases ΔK saves uplink bandwidth without affecting classification accuracy.

A linear scoring head maps each token from d=768 to a scalar importance $\pi_{l}$. To select exactly $K_{u}$ tokens while maintaining gradient flow during training, we use Gumbel-Top*K* with a straight-through estimator:

(12) $$m=\underset{\mathrm{stop}\;\mathrm{gradient}}{\underset{︸}{\left(m_{\mathrm{hard}}-\mathrm{softmax}\left(y\right)\right)}}+\mathrm{softmax}\left(y\right),$$

where y=π+g with g∼Gumbel(0,1), and $m_{\mathrm{hard}}\in {\left\{0,1\right\}}^{L}$ is a binary mask whose top-$K_{u}$ entries are set to 1. In the forward pass the hard mask determines which tokens are retained; in the backward pass gradients propagate through the softmax surrogate.

The $K_{u}$ selected tokens are projected from d=768 to a bottleneck dimension $d_{b}=8$ via a linear layer and quantized to q=4 bits, yielding the transmitted set

(13) $$Z_{\mathrm{tx}}=\left\{\mathrm{proj}\left({\tilde{z}}_{l}\right)|m_{l}=1\right\}\in R^{K_{u}\times d_{b}},$$

with a per-sample uplink payload of $K_{u}\times b_{\mathrm{token}}$ bits ($b_{\mathrm{token}}=d_{b}\times q=32$). Because the hard clip enforces $K_{u}\leq K_{\max}^{\left(t\right)}$, the payload fits the OFDM slot capacity under every channel realization.

During training the selected tokens are aggregated into a single fixed-length representation through differentiable weighted pooling:

(14) $$z=\sum_{l=1}^{L}\alpha_{l}\;{\tilde{z}}_{l},\;\alpha =\mathrm{softmax}\left(\mathrm{gate}\left(\tilde{Z}\right)⊙m\right),$$

where gate(·) is a linear projection producing a scalar relevance score per token. The mask m zeros out unselected entries so that only the $K_{u}$ retained tokens contribute to the pooled vector z. At deployment the server receives the $K_{u}$ projected tokens over the uplink and applies the same gated pooling to form the input for the task heads.

### 4.3. Computational Overhead

Table 2 reports the computational cost of the SGAM module measured in multiply–accumulate operations (MACs). The total SGAM overhead is $6.4\times 10^{5}$ MACs, representing only 0.014% of the backbone cost ($4.5\times 10^{9}$ MACs). This translates to approximately 0.06 ms per sample on a single edge GPU—negligible compared with the 3.5 ms backbone latency. On the server side, CDFL’s per-round cost for pairwise RBF similarity computation ($O(U^{2})$) and facility location selection totals below 0.1 ms for U=20 clients. For fleets exceeding U≈1000, we implement a two-stage approximation that reduces this to O(UlogU), as detailed in Section 8.6.

## 5. CDFL: Channel-Decoupled Client Selection

SGAM ensures that a selected vehicle’s output fits the channel, but does not address *which* vehicles should participate. As noted in Section 1, utility-based scheduling conflates channel quality with data value, systematically excluding the vehicles whose data matters most. CDFL tackles this by decoupling the two dimensions.

### 5.1. Quadrant Decomposition

Each client *i* is characterized by a four-dimensional descriptor $c_{i}=\left[{\mathrm{SNR}}_{i},\;\kappa_{i},\;\ell_{i},\;H_{i}\right]$, where ${\mathrm{SNR}}_{i}$ is the instantaneous signal-to-noise ratio measured at the RSU, $\kappa_{i}$ is the Rician *K*-factor in linear scale (a proxy for line-of-sight quality, where $\kappa_{i}=1$ corresponds to 0 dB, i.e., equal LOS/NLOS power, and $\kappa_{i}\gg 1$ indicates a dominant line-of-sight component), $\ell_{i}$ is the local training loss from the previous round, and $H_{i}=-\sum_{c}p_{c}\log p_{c}$ is the Shannon entropy of the local label distribution ${\left\{p_{c}\right\}}_{c=0}^{3}$, where $p_{c}$ is the fraction of local samples belonging to class *c*. The first two components summarize the communication link; the latter two summarize the statistical complexity of the local data.

Two composite indices are computed from per-round medians:

(15) $${\mathrm{ChQ}}_{i}=1\left\{{\mathrm{SNR}}_{i}\geq \mathrm{med}\left(\mathrm{SNR}\right)\right\}+1\left\{\kappa_{i}\geq \mathrm{med}\left(\kappa \right)\right\},$$

(16) $${\mathrm{SemD}}_{i}=1\left\{\ell_{i}>\mathrm{med}\left(\ell \right)\right\}+1\left\{H_{i}<\mathrm{med}\left(H\right)\right\}.$$

Both take values in {0,1,2}. Binarizing at threshold 1 (${\mathrm{ChQ}}_{i}\geq 1$: good channel; ${\mathrm{SemD}}_{i}\geq 1$: high complexity) yields four quadrants.

$Q_{1}$ (poor channel, complex data) contains vehicles behind buildings at busy intersections—poor propagation, dense occluded scenes, high training value but the first to be excluded by throughput-based rules. $Q_{2}$ (poor channel, simple data) holds vehicles with weak links and undemanding scenes, for example a vehicle in a parking structure viewing an empty lot. $Q_{3}$ (good channel, complex data) represents the ideal case: strong links and rich data; all scheduling strategies tend to include these. $Q_{4}$ (good channel, simple data) covers open-road vehicles whose high throughput inflates their utility score despite low data informativeness.

In our experiments with the Oort-V2X baseline, $Q_{1}$ vehicles were selected in only 8% of rounds while $Q_{4}$ vehicles were selected in 42%.

### 5.2. Facility Location Selection

A minimum quota $N_{j}$ is enforced per quadrant, with $\sum_{j}N_{j}=K$ (the total number of clients selected per round). Within each quadrant, representatives are chosen by solving a facility location problem over a radial basis function (RBF) similarity kernel:

(17) $$\mathrm{sim}\left(i,j\right)=\exp\;\left(-\frac{∥c_{i}-c_{j}{∥}^{2}}{2\sigma^{2}}\right),$$

where $c_{i}$ and $c_{j}$ are the multi-dimensional descriptors for clients *i* and *j* as defined above, and σ is the bandwidth parameter of the RBF kernel that controls sensitivity to descriptor differences. We set σ=1.0 throughout. The selection objective for quadrant *j* is

(18) $$\max_{A_{j}\subset Q_{j},\;\left|A_{j}\right|=N_{j}}\;\sum_{u\in Q_{j}}\max_{v\in A_{j}}\mathrm{sim}\left(c_{u},c_{v}\right).$$

Because this function is monotone submodular, a greedy algorithm that iteratively adds the client with the largest marginal gain yields a (1−1/e) approximation to the optimum in $O(|Q_{j}|\cdot N_{j})$ time—negligible compared with local model training. The greedy selection spreads the chosen clients across the feature space of each quadrant rather than clustering them at one operating point, providing inter-quadrant coverage (all channel–data conditions represented) together with intra-quadrant diversity (no redundant selections within a quadrant).

### 5.3. Inverse Propensity Aggregation

Enforcing per-quadrant quotas introduces a sampling bias: clients in smaller quadrants are selected at a higher rate relative to their population share. To prevent this from distorting the aggregated model, we apply inverse propensity weighting (IPW) during global model aggregation [29]:

(19) $$w_{i}=\frac{{\left(|Q\left(i\right)|+\varepsilon \right)}^{-\alpha }}{\sum_{j\in A}{\left(|Q\left(j\right)|+\varepsilon \right)}^{-\alpha }},$$

where Q(i) denotes the quadrant containing client *i*, ε=1.0 prevents division by zero if a quadrant is empty, and α∈[0,1] controls the correction strength (α=0: uniform weights; α=1: full compensation). We set α=0.5 throughout. The global model update is

(20) $$\theta^{\left(t+1\right)}=\frac{\sum_{u\in A}w_{u}D_{u}\theta_{u}^{\left(t\right)}}{\sum_{u\in A}w_{u}D_{u}},$$

where $D_{u}$ is the size of client *u*’s local dataset and $\theta_{u}^{\left(t\right)}$ is the locally updated model.

The combined effect of quadrant quotas and IPW is that $Q_{1}$ vehicles—those with the poorest channels and the most safety-critical data—receive a larger share of influence on the global update than their population size alone would warrant. In our experiments CDFL raised the effective selection rate of $Q_{1}$ from 8% (under Oort-V2X) to 22%. The ablation study in Section 8 shows that removing IPW while retaining the quadrant structure costs 5.5 Macro-F1 points, confirming that both stratified coverage and propensity correction are necessary.

## 6. Training Objective

The per-client loss has three groups of terms: task-specific losses, a sensing regularizer, and system-level constraints.

### 6.1. Task Losses

The primary task is scene complexity classification into four ordered classes. Because the classes carry an ordinal structure—misclassifying an empty road as a complex intersection wastes uplink spectrum on an unnecessary cooperative perception request, while the reverse suppresses a safety-critical transmission—we augment standard soft cross-entropy with an Earth Mover’s Distance (EMD) penalty that significantly increases the cost of predictions compared to the true class in the ordinal sense:

(21) $$L_{\mathrm{main}}=L_{\mathrm{softCE}}+\lambda_{\mathrm{EMD}}\;L_{\mathrm{EMD}},$$

with $\lambda_{\mathrm{EMD}}=0.20$. The soft cross-entropy uses Gaussian-smoothed labels (σ=1.0) to reduce overfitting on small local datasets.

Two auxiliary heads share the backbone representation: three independent binary classifiers for object presence, each predicting the existence of one category (vehicle, pedestrian, or cyclist), with loss $L_{\mathrm{aux}}$, and a three-dimensional regression head for object counts with loss $L_{\mathrm{count}}$. Neither output is transmitted over the uplink; both serve solely to regularize the shared feature space during training. The combined task loss is

(22) $$L_{\mathrm{comm}}=L_{\mathrm{main}}+\lambda_{\mathrm{aux}}L_{\mathrm{aux}}+\lambda_{\mathrm{count}}L_{\mathrm{count}}+\lambda_{H}H\left(p\right),$$

with $\lambda_{\mathrm{aux}}=0.30$, $\lambda_{\mathrm{count}}=0.10$, and an entropy term $\lambda_{H}=0.01$ that discourages excessively uncertain predictions.

### 6.2. Sensing Regularizer

In preliminary experiments the encoder largely ignored the sensing components of $q_{u}$ (range, velocity, sensing confidence): the FiLM parameters γ and β converged to near-identity values, effectively bypassing the physical-layer side information. To encourage the model to exploit these inputs when they carry useful information, we added a CRB-derived regularizer:

(23) $$L_{\mathrm{sens}}=\mathrm{clip}\;\left(\log\;\left(1+\frac{\mathrm{tr}(W\cdot \mathrm{CRB})}{\mathrm{EMA}(\mathrm{tr}(W\cdot \mathrm{CRB}))}\right),\;0,\;\tau \right),$$

where W is a learnable diagonal weight matrix that lets the model discover which sensing dimensions (range, velocity, angle) are most informative for different scene types, EMA denotes an exponential moving average that provides a stable baseline to reduce loss variance, and τ=5.0 clips the output to prevent extreme values during early training. The regularizer is small when CRB is small (precise sensing) and grows as sensing degrades, encouraging the encoder to rely more heavily on the condition vector.

Removing this regularizer in the ablation study (Section 8) reduces Macro-F1 from 0.710 to 0.690 and worst-class F1 from 0.465 to 0.410—the smallest effect among all ablated components, consistent with sensing information serving as a supplementary rather than primary training signal.

### 6.3. Total Loss

The task loss $L_{\mathrm{comm}}$ and the sensing regularizer $L_{\mathrm{sens}}$ operate on different scales and exhibit different noise levels. We balance them through learned homoscedastic uncertainty weighting [30]:

(24) $$L_{\mathrm{UW}}=\frac{L_{\mathrm{comm}}}{2\sigma_{c}^{2}}+\frac{L_{\mathrm{sens}}}{2\sigma_{s}^{2}}+\log\left(\sigma_{c}\sigma_{s}\right),$$

where $\sigma_{c}$ and $\sigma_{s}$ are learnable scalar parameters that remove the need for manual tuning of the relative weight between task and sensing losses: a high-variance component drives its σ up, automatically reducing its influence, while the logarithmic penalty prevents σ from growing without bound.

The final per-client training loss adds two system-level regularizers:

(25) $$L=L_{\mathrm{UW}}+\lambda_{\mathrm{over}}\;\mathrm{ReLU}\left(K_{u}-K_{\max}^{\left(t\right)}\right)+\frac{\mu }{2}{∥\theta -\theta^{\left(t\right)}∥}^{2}.$$

The first term penalizes any token count exceeding the slot budget ($\lambda_{\mathrm{over}}=1.0$). In practice this penalty never activates because the hard clip in Equation (11) already prevents $K_{u}>K_{\max}^{\left(t\right)}$; the term is retained as a numerical safeguard during early training before the ΔK MLP has converged. The second term is the FedProx proximal penalty [6] with μ=0.05, which limits local model drift across heterogeneous clients.

## 7. Convergence Analysis

We provide a convergence bound for the proposed framework under standard non-convex FL assumptions, following the analytical structure of [6,7].

**Assumption** **1.** *Three standard conditions are adopted: (A1) the global loss L(w) is L-smooth, i.e. ${∥\nabla L\left(w\right)-\nabla L\left(v\right)∥}_{2}\leq L{∥w-v∥}_{2}$; (A2) the variance in each client’s stochastic gradient is bounded by $\sigma^{2}$; (A3) client heterogeneity is bounded according to $∥\nabla L_{u}{\left(w\right)-\nabla L\left(w\right)∥}_{2}^{2}\leq \Gamma^{2}$ for all u∈U.*

**Lemma** **1** (Gradient error under CDFL)**.** *Let $A_{t}$ denote the K clients selected at round t and $g_{A_{t}}=\frac{1}{K}\sum_{u\in A_{t}}\nabla L_{u}\left(w_{t}\right)$ represent the aggregated gradient. Under quadrant-stratified facility location selection,*

(26) $$E\;\left[∥g_{A_{t}}-\nabla L\left(w_{t}\right){∥}_{2}^{2}\right]\leq \frac{\Gamma^{2}}{K}\left(1-\delta_{\mathrm{sub}}\right)+\frac{\sigma^{2}}{K},$$

*where $\delta_{\mathrm{sub}}=1-1/e$ is the greedy approximation ratio for monotone submodular maximization.*

The hypothesis is that high coverage in the client descriptor space $c_{i}$ reduces the net bias of the aggregated gradient. This connection holds when the mapping from client descriptors to local gradient directions is Lipschitz continuous. We treat this as a working assumption. The variance reduction observed in Section 8 is consistent with it, although a formal proof under weaker conditions is left for future work.

**Theorem** **1.** *With step size $\eta_{t}=\eta_{0}/\sqrt{t+1}$ and under (A1)–(A3) together with Lemma 1, the iterates satisfy*

(27) $$\frac{1}{T}\sum_{t=0}^{T-1}E\;\left[∥\nabla L\left(w_{t}\right){∥}_{2}^{2}\right]\leq \frac{2\Delta }{\eta_{0}\sqrt{T}}+\frac{C_{1}\sigma^{2}}{\sqrt{T}}+\frac{C_{2}\Gamma^{2}\left(1-\delta_{\mathrm{sub}}\right)}{\sqrt{T}},$$

where $\Delta =L\left(w_{0}\right)-L^{*}$ and $C_{1}=C_{2}=L/2$.

**Proof.**  By *L*-smoothness each update satisfies $L\left(w_{t+1}\right)\leq L\left(w_{t}\right)-\eta_{t}\langle \nabla L\left(w_{t}\right),g_{t}\rangle +\frac{L\eta_{t}^{2}}{2}{∥g_{t}∥}^{2}$. Taking expectations and substituting the bound from Lemma 1 gives $E\left[L\left(w_{t+1}\right)\right]\leq E\left[L\left(w_{t}\right)\right]-\frac{\eta_{t}}{2}E[∥\nabla L\left(w_{t}\right){∥}^{2}]+\frac{L\eta_{t}^{2}}{2K}\left[\sigma^{2}+\Gamma^{2}\left(1-\delta_{\mathrm{sub}}\right)\right]$. Telescoping over *T* rounds with $\eta_{t}=\eta_{0}/\sqrt{t+1}$ and using $\sum \eta_{t}\geq \eta_{0}\sqrt{T}$, $\sum \eta_{t}^{2}\leq \eta_{0}^{2}\left(1+\ln T\right)$ yields the stated bound.    □

All three terms decay as $O(1/\sqrt{T})$. The effect of CDFL appears in the third term, where the heterogeneity coefficient $\Gamma^{2}$ is multiplied by $(1-\delta_{\mathrm{sub}})\approx 0.368$. Note that the bound does not model the FedProx proximal term or the budget-enforcement regularizer in Equation (25); both further improve convergence in practice. The theoretical step-size schedule also differs from the constant learning rate used in experiments, so the bound should be interpreted as qualitative guidance rather than a quantitative prediction.

## 8. Experiments

### 8.1. Setup

**Dataset.** We use the NuScenes v1.0-trainval dataset (850 driving scenes, Boston and Singapore, day/night, rain/clear) [31]. Key frames are extracted from the front-facing camera (CAM_FRONT) and partitioned among U=20 clients via a Dirichlet distribution with concentration α=0.5 to produce non-IID label distributions. Scene complexity labels are obtained by quantile-binning per-frame object density and occlusion ratio into four balanced classes: empty (Class 0), low (Class 1), medium (Class 2), and high (Class 3). The NuScenes v1.0-trainval dataset is publicly available at https://www.nuscenes.org/nuscenes (accessed on 20 March 2026) upon free registration. Our data preparation and channel simulation scripts will be released as open-source code upon acceptance.

**Channel model.** Carrier frequency $f_{c}=5.9$ GHz, following 3GPP NR V2X evaluation methodology. Per-client instantaneous SNR covers the [5,25] dB range to capture propagation diversity from NLOS urban canyons to LOS suburban corridors. The Rician *K*-factor varies from 0 dB (equal LOS/NLOS power) to 10 dB (strong LOS). Subcarrier allocation uses a proportional fair scheduler with orthogonal assignment across vehicles.

**OFDM parameters.** $N_{\mathrm{sc}}=128$ subcarriers, $N_{\mathrm{sym}}=14$ symbols per slot, 16-QAM (M=16), coding rate $R_{c}=0.5$, pilot overhead $\rho_{\mathrm{pilot}}=0.10$, cyclic prefix ratio $\rho_{\mathrm{cp}}=0.07$. Semantic tokens use bottleneck dimension $d_{b}=8$ with 4-bit quantization, giving $b_{\mathrm{token}}=32$ bits per token.

**Model.** ConvNeXt-V2-Tiny pretrained on ImageNet-22k; stages 0–2 frozen, stage 3 trainable. The backbone produces L=49 spatial tokens with d=768. FiLM MLP architecture: [6,256,1536] with GELU. Token scoring head: Linear (768,1).

**Training.** 60 FL rounds, 2 local epochs per round, batch size 8, AdamW optimizer (learning rate $10^{-4}$, weight decay $10^{-4}$), gradient clipping at norm 1.0, FedProx coefficient μ=0.05, 10 clients selected per round.

**Baselines.** All methods share the same ConvNeXt-V2-Tiny backbone. Baselines transmit a fixed $K_{\mathrm{fixed}}=32$ tokens per frame (the median of the dynamic $K_{\max}$ range in our channel simulation) and use standard cross-entropy loss without ordinal awareness, uncertainty weighting, or entropy regularization.

- **FedAvg** [14]: uniform random client selection, fixed-*K* transmission.
- **FedProx** [6]: proximal regularization (μ=0.01), fixed-*K* transmission. We use μ=0.01 following the original paper’s recommended range; the proposed method uses μ=0.05 because the additional SGAM and CDFL components alter the loss landscape and benefit from slightly stronger drift control, as confirmed by the sensitivity analysis in Section 8.5.
- **Oort-V2X** [3]: V2X-adapted utility $U_{i}=\left|L_{i}\right|\times \sqrt{{\mathrm{SNR}}_{i}}$, selecting clients that maximize this product.

**Remark on baseline provenance.** FedAvg [14], FedProx [6], and Oort [3] are published by AISTATS, MLSys, and USENIX OSDI—venues that serve as primary outlets for systems-oriented machine learning research and are widely regarded as top-tier in the computer science community. These three methods constitute the standard baselines in the federated learning literature. To strengthen the comparison with communication-oriented work, we additionally survey two recent IEEE Transactions papers that address resource-adaptive semantic communication in vehicular networks—Su et al. [13] and Wang et al. [10]—and position them against the proposed framework in the research gap analysis (Table 1, Section 2.4). A direct experimental comparison with [13] would require adapting their Lyapunov-based centralized D2D power control to the multi-client FL training loop, which constitutes a non-trivial engineering effort; we leave this integration for future work.

### 8.2. Main Results

Table 3 compares all methods after 60 rounds, and Figure 3 shows the corresponding convergence trajectories.

After 60 rounds our method reaches 0.710 Macro-F1, 8.7 points above Oort-V2X and 19.0 points above FedAvg. In the convergence curves all three baselines maintain wide confidence bands throughout training. Oort-V2X oscillates visibly in rounds 15–25; these oscillations coincide with rounds where several $Q_{1}$ vehicles experienced deep fading and were excluded from the selected pool, temporarily removing complex-scene data from the training process. The proposed method narrows to a tight band after roughly round 30.

Training variance drops sharply: *σ*_last-10_ goes from 3.2% (FedAvg) to 0.8%, largely because SGAM removes truncation events. Without it, 23% of transmissions exceed slot capacity and the corrupted gradients compound over rounds.

The accuracy gain is largely due to CDFL raising $Q_{1}$ selection from 8% to 22%, thereby feeding complex-scene gradients into every round. Worst-class F1 goes from 0.215 to 0.465—a classifier that misses complex intersections either creates safety blind spots or wastes spectrum on unnecessary cooperative transmissions.

### 8.3. Ablation Study

Table 4 isolates the contribution of each component by removing one at a time from the full framework.

Dropping SGAM entirely causes the largest degradation: accuracy falls to 62.1% and training variance rises to 4.1%, both worse than FedAvg. The system reverts to a fixed K=32 output with a 23% budget-violation rate; each violation corrupts the transmitted features and the resulting biased gradients accumulate over rounds. Removing only the hard budget clip while keeping the scoring network and FiLM still produces over 20% violations and a 4.9-point accuracy loss relative to the full model, confirming that soft token ranking cannot substitute for a hard capacity constraint.

Without FiLM conditioning, overall accuracy drops by 3.1 points but worst-class F1 falls more sharply, from 0.465 to 0.310. Per-class analysis traces the degradation to high-speed NLOS scenes (v>30 m/s) where Doppler blur corrupts texture-related feature channels. FiLM learns to suppress these channels and rely on structural features instead; without it the encoder treats all feature dimensions equally regardless of their reliability under the current propagation conditions.

Removing CDFL costs 9.5 Macro-F1 points and increases variance to 2.8%. The framework falls back to Oort-level scheduling and $Q_{1}$ vehicles are selected in only 8% of rounds, so the model loses steady exposure to complex-scene data. Removing IPW alone while keeping the quadrant structure costs a further 5.5 points, showing that both stratified coverage and propensity correction contribute.

The sensing regularizer has the smallest marginal effect among all components: Macro-F1 drops by 2.0 points and worst-class F1 by 5.5 points. This suggests that ISAC-derived parameters act mainly as a secondary signal, helping on difficult scenes while contributing little on easy ones.

### 8.4. Resource Compliance and Client Coverage

**Figure 4:**
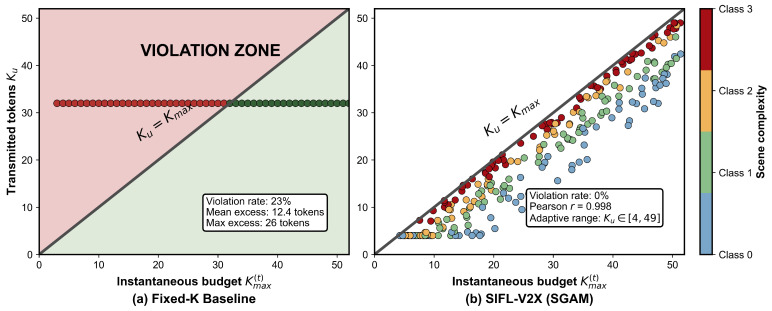
Transmitted tokens vs. budget across all 600 selected client-round pairs. **Left**: fixed-K=32 baseline (23% violations). **Right**: SGAM (0% violations, Pearson r=0.998).

Figure 4 plots the transmitted token count $K_{u}$ against the instantaneous budget $K_{\max}^{\left(t\right)}$ across all 600 selected client-round pairs (10 active clients per round × 60 rounds).

With the fixed-*K* baseline, 23% of transmission instances fall above the diagonal—the encoder attempted to send more tokens than the slot could carry and the excess was lost. With SGAM every point sits on or below the diagonal; the Pearson correlation between $K_{u}$ and $K_{\max}^{\left(t\right)}$ is 0.998. The hard clip in Equation (11) ensures that no transmission can exceed the slot budget. The average uplink payload per client per round drops by 38% relative to the fixed-K=32 baseline at equivalent classification accuracy, because the encoder no longer wastes capacity on low-budget slots and because the learned ΔK further reduces the token count for simple scenes that do not need the full allocation.

Table 5 reports client selection frequency by quadrant. Under Oort-V2X the ratio between $Q_{1}$ and $Q_{4}$ selection rates exceeds 1:5. Under the proposed framework all four quadrants fall within the 22–27% range, close to the uniform 25% expected if the quadrants were equally sized.

### 8.5. Hyperparameter Sensitivity

**Figure 5:**
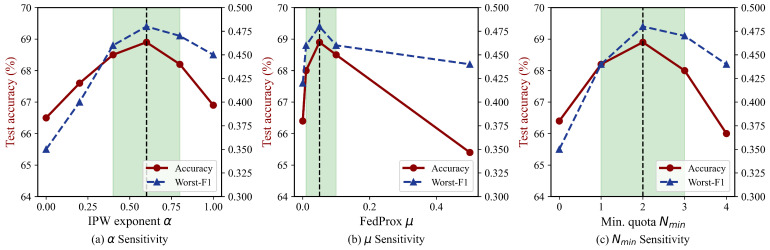
Sensitivity to (**a**) α, (**b**) μ, and (**c**) $N_{\min}$. Shaded regions indicate ≤1 pp deviation from the peak.

Figure 5 sweeps three scheduling hyperparameters while holding the remaining two at their defaults (α=0.5, μ=0.05, $N_{\min}=2$).

Performance is stable over α∈[0.3,0.8], with a gentle peak near α=0.5; beyond 0.8 the over-correction amplifies noise from the small $Q_{1}$ datasets. The FedProx coefficient works well across μ∈[0.01,0.1]: below 0.01 the proximal term is too weak to prevent drift under strong non-IID conditions, while above 0.1 it over-constrains local updates and slows convergence. For the per-quadrant quota, accuracy improves from $N_{\min}=0$ (quadrant structure defined but not enforced) to $N_{\min}=2$, then plateaus through $N_{\min}=3$; at $N_{\min}=4$ each quadrant must supply 4 of the 10 selected clients, which limits intra-quadrant diversity.

Across all three sweeps, both accuracy and worst-class F1 remain within 1% of the peak over at least half of the tested range, indicating that the framework is not sensitive to precise hyperparameter settings.

### 8.6. Scalability and End-to-End Latency Analysis

**Scalability of CDFL.** The exact facility location selection in Section 5 incurs $O(U^{2})$ cost from pairwise RBF similarity evaluations, which is negligible at U=20 but grows prohibitive for large fleets. We implement a two-stage approximation to reduce the overall scheduling complexity to O(UlogU).

*Stage 1—LSH graph construction.* We build a sparse *k*-nearest-neighbor graph over the client descriptors $\left\{c_{i}\right\}$ using Locality-Sensitive Hashing (LSH) with p=⌈2logU⌉ random projections tuned to the RBF bandwidth σ. Each client’s candidate set is restricted to its k=⌈logU⌉ approximate nearest neighbors, reducing per-query cost from O(U) to O(logU). Graph construction costs O(UlogU) in total.

*Stage 2—Stochastic Greedy selection.* Rather than evaluating all candidates at each greedy step, we sample a random subset of size $\frac{U}{K}\log\left(1/\epsilon \right)$ following [32] and restrict similarity evaluations to the LSH neighbor sets. The combined per-step cost is $O\;\left(\frac{U}{K}\log\left(1/\epsilon \right)\cdot \log U\right)$, giving an overall complexity of $O\;\left(U\log U\cdot \log(1/\epsilon )\right)$, i.e., O(UlogU) for fixed ϵ.

To validate this, we conducted a synthetic scalability test by generating client descriptor sets of size up to U=5000 and measuring wall-clock time on a standard CPU (Intel Core i9-12900K, Intel Corporation, Santa Clara, CA, USA). As shown in Table 6, at U=1000 the exact algorithm requires 45.2 ms, whereas the LSH-augmented Stochastic Greedy approximation requires only 4.2 ms, retaining 98.5% of the optimal facility location objective value. At U=5000 the approximation completes in 21.6 ms. These results confirm that CDFL scales to dense urban deployments without becoming a scheduling bottleneck.

**End-to-End Latency.** V2X cooperative perception mandates end-to-end latency below 100 ms [25]. We now account for every major latency component of the proposed framework.

On the vehicle side, the ConvNeXt-V2-Tiny backbone requires approximately 3.5 ms, and the SGAM module (FiLM conditioning, token scoring, Gumbel-TopK, and linear projection) adds only 0.06 ms (Table 2), giving a total on-vehicle processing time of ≈3.6 ms.

For uplink transmission, the adaptive semantic payload averages 28tokens×32bits/token=896 bits per vehicle. Under 5G NR V2X numerology with 15 kHz subcarrier spacing, one transmission slot spans exactly 1 ms. Because the orthogonal subcarrier assignment ($S_{u}\cap S_{v}=\emptyset$) allows all K=10 selected vehicles to transmit simultaneously within the same slot, the fleet-level uplink latency is 1 ms.

On the server side, the approximate CDFL scheduling for U=1,000 clients requires 4.2 ms (Table 6). Subsequent weighted model aggregation (Equation (20)) adds less than 1 ms for the ConvNeXt-V2-Tiny parameter count.

The total per-round latency—vehicle processing (3.6 ms), simultaneous uplink transmission (1 ms), and server scheduling (4.2 ms)—is therefore **under 10 ms**, well within the 100 ms V2X real-time constraint and leaving ample margin for downstream cooperative fusion and global model aggregation.

## 9. Discussion

**Channel model scope.** The channel simulation follows 3GPP NR V2X statistical methodology with path loss, log-normal shadowing, Rician fading, and proportional fair scheduling. This captures the defining feature of vehicular OFDM—slot-level resource volatility—but omits hardware-specific effects such as power-amplifier nonlinearity, carrier frequency offset, and quantized CSI feedback. These impairments would reduce the precision of the budget estimate in Equation (6) and, consequently, the token budget in Equation (7). The hard clip in Equation (11) provides a degree of robustness because it enforces a strict upper bound regardless of estimation error, but validating this robustness on a software-defined radio testbed with over-the-air V2X transmission remains necessary.

**Impact of imperfect CSI.** In practice, channel estimation error introduces a mismatch between the estimated and true channel states. However, CSI imperfection does not compromise the budget-compliance guarantee of SGAM. The token budget $K_{\max}^{\left(t\right)}$ is computed from the actual subcarrier set $S_{u}^{\left(t\right)}$ explicitly allocated by the RSU and communicated to each vehicle through downlink control signaling; it is not derived from the vehicle’s local channel estimate. Therefore, the semantic violation rate remains 0% regardless of estimation error.

What CSI error does affect is physical-layer reliability and scheduling optimality. If the RSU overestimates the channel quality, the fixed 16-QAM modulation may suffer from increased block error rate, degrading effective throughput. Furthermore, the condition vector $q_{u}$ used for FiLM modulation will contain a noisy SNR estimate. To quantify these combined effects, we injected multiplicative error ${\hat{h}}_{u,k}=h_{u,k}\left(1+\epsilon \right)$ with $\epsilon ∼\mathrm{CN}(0,\sigma_{e}^{2})$. As shown in Table 7, performance degrades gracefully. At 10% NMSE ($\sigma_{e}^{2}=0.10$), Macro-F1 drops by 2.1 points, primarily impacting Worst-F1 (−4.5 points) since complex-scene vehicles in NLOS conditions are most vulnerable to scheduling suboptimality. Yet, the proposed method (0.689) still substantially outperforms the perfect-CSI Oort baseline (0.623), confirming robustness under realistic channel estimation conditions.

**Sensitivity to subcarrier count.** To verify that the framework generalizes across bandwidth configurations, we evaluated the system with $N_{\mathrm{sc}}\in \left\{64,128,256\right\}$. Table 8 reports the results. Across all configurations, the hard clip in Equation (11) prevents every budget violation. A larger $N_{\mathrm{sc}}$ yields a wider $K_{\max}$ range, giving SGAM more headroom to allocate tokens to complex scenes. At $N_{\mathrm{sc}}=64$, the minimum observed $K_{\max}$ drops to 4; nevertheless, the Macro-F1 of 0.676 still exceeds the Oort-V2X baseline (0.623). When $N_{\mathrm{sc}}=256$, the upper bound of $K_{\max}$ is capped at 49 by the architectural ceiling $k_{\mathrm{cap}}=L=49$ rather than by the channel capacity limit, indicating that at this bandwidth the encoder operates near its maximum token capacity on good-channel slots. The framework does not require retraining when $N_{\mathrm{sc}}$ changes; only the resource mapper parameter in Equation (6) updates dynamically.

**Failure modes.** We identify three conditions under which the framework degrades significantly.

*(1) Sensing confidence collapse.* When the ISAC echo is blocked entirely—for instance, by a large vehicle between the RSU and the target—the sensing confidence $\xi_{u}$ in Equation (5) drops to near zero. In this regime the FiLM parameters γ and β converge toward identity values, effectively bypassing the sensing input. The encoder falls back to backbone-only features, which corresponds to the operating point of the “w/o sens loss” ablation variant (Table 4: Macro-F1 = 0.690, Worst-F1 = 0.410). The system does not fail catastrophically but loses the benefit of sensing-guided feature adaptation.

*(2) Extreme non-IID with empty quadrants.* Under very aggressive non-IID conditions (Dirichlet α<0.1), some quadrants may be empty in certain rounds, forcing CDFL to fall back to random selection for the missing quota and partially negating the fairness benefit. Adaptive quadrant boundaries using exponential moving averages of the per-round medians could mitigate this.

*(3) Rapidly varying topology beyond coherence time.* If vehicle speed exceeds the range where the proportional fair scheduler can track the channel, both the subcarrier allocation and the FiLM conditioning become stale. The hard clip still prevents violations, but the ΔK adjustment and FiLM modulation lose accuracy, degrading Macro-F1. To overcome this reactive limitation, integrating predictive generative architectures offers a promising future direction. Recent advances have demonstrated that world models can effectively capture the long-term dynamics of wireless environments [33] and optimize latency-critical metrics in vehicular networks [34]. Augmenting our framework with a world model could enable proactive semantic token budgeting before channel state degradation occurs.

**Trade-offs and broader considerations.**

*(1) Computational cost vs. bandwidth savings.* The SGAM overhead of 0.06 ms per sample (Table 2) is negligible relative to the 3.5 ms backbone latency. The 38% bandwidth reduction translates to approximately 12 Mbps of freed uplink capacity per RSU in a 20-vehicle, 10 Hz deployment—enough to support 3–4 additional vehicles.

*(2) Comparison with rate-distortion approaches.* Classical variable-rate compression [12] optimizes a continuous rate–distortion trade-off by adjusting the bottleneck prior. SGAM differs in that the target metric is downstream classification accuracy rather than reconstruction fidelity, and the rate constraint is a hard, externally imposed slot capacity that changes every transmission interval rather than a soft Lagrangian penalty. A direct comparison is difficult because rate-distortion methods require retraining for each target rate, whereas SGAM handles arbitrary rates within a single model through the Gumbel-TopK mechanism.

*(3) Privacy.* The federated architecture provides inherent privacy protection: raw sensor images never leave the vehicle. The CDFL descriptor $c_{i}=\left[{\mathrm{SNR}}_{i},\kappa_{i},\ell_{i},H_{i}\right]$ does require the server to observe per-client training loss and label entropy, which reveals coarse distributional information about local data. In applications with strict privacy requirements, the descriptor could be protected through local differential privacy at the cost of noisier quadrant assignment.

**Applicability to UAV-assisted V2X.** Although our experiments use a ground-based RSU, the proposed framework is designed to operate whenever the uplink resource allocation fluctuates across slots—a condition that is at least as pronounced in UAV-to-vehicle links. First, UAV altitude and attitude changes cause rapid variation in the air-to-ground channel, producing slot-level subcarrier reassignment that SGAM’s hard-clip mechanism absorbs without modification. The altitude-dependent Rician *K*-factor in such deployments makes the adaptive token budgeting of SGAM particularly valuable [8]. Second, the compact semantic representation transmitted by SGAM (averaging 28 tokens × 32 bits = 896 bits per frame) directly reduces the energy consumed by the UAV’s communication subsystem. Compared to standard fixed-*K* transmission at equivalent accuracy, SGAM yields a 38% reduction in uplink payload, which is vital for extending mission endurance. Third, the spatial correlation between channel quality and scene complexity that CDFL is designed to counteract persists in UAV-V2X: vehicles near the UAV’s nadir typically observe simpler scenes than vehicles at the coverage boundary near intersections. Quantitative evaluation under realistic UAV mobility models constitutes a promising direction for future work.

**Task and scale limitations.** The evaluation task is a four-class scene-complexity classifier that acts as a semantic gate for cooperative perception. Extending the framework to spatially resolved tasks such as bird’s-eye-view segmentation or 3D object detection would require replacing the global pooling in Equation (14) with a mechanism that preserves the spatial layout of the selected tokens—sparse attention over retained positions is one candidate, but its interaction with token pruning has not been studied. On the scalability side, the pairwise similarity computation in Equation (17) is $O(U^{2})$, which is negligible at U=20 but would require the LSH-augmented Stochastic Greedy approximation detailed in Section 8.6 to be used beyond U≈1000, as is empirically validated in Table 6. Whether the four-quadrant partition remains the right granularity at larger fleet sizes or whether a finer grid is needed is an open question.

**Convergence analysis.** The bound in Theorem 1 relies on an assumption (implicit in Lemma 1) that high coverage in the client descriptor space translates to reduced gradient bias. While the experimental variance reduction supports this hypothesis, a rigorous proof would need to establish a Lipschitz mapping between descriptor space and gradient space, which we leave for future work. In addition, the theoretical step-size schedule ($\eta_{t}\propto 1/\sqrt{t}$) differs from the constant AdamW rate used in practice; the bound is therefore best read as qualitative guidance on the benefit of coverage-maximizing selection rather than a tight quantitative prediction.

## 10. Conclusions

This work addressed the resource–rate mismatch and the channel–data scheduling bias that arise together in OFDM-based V2X semantic communication.

On the transmission side, SGAM converts each slot’s resource-block allocation into a hard token budget enforced by a capacity clip, which prevents truncation events during transmission and reduces training variance from 3.2% to 0.8%. The average uplink payload drops by 38% at equal classification accuracy because the encoder no longer wastes capacity on slots that cannot support the full token count and further reduces its output for scenes that do not need it.

On the scheduling side, CDFL partitions clients according to channel quality and data complexity, selects diverse representatives per partition through facility location optimization, and corrects for partition-size imbalance via inverse propensity weighting. The selection rate of the most underserved vehicle group ($Q_{1}$) rises from 8% to 22%, driving an 8.7-point Macro-F1 gain over the Oort baseline and more than doubling worst-class F1 relative to FedAvg.

The channel model omits hardware impairments, as the task does not yet extend beyond four-class classification, and scalability beyond 20 vehicles has not been experimentally validated under full FL training—although the scheduling approximation analysis in Section 8.6 confirms that the computational overhead of CDFL scales gracefully to U=5000 clients. In future work we plan to validate the framework on a hardware testbed with over-the-air V2X transmission, extend the token-selection mechanism to LiDAR–camera cross-modal fusion, and explore spatially selective ISAC integration in which sensing-derived target locations guide token allocation across the feature map.

## Figures and Tables

**Table 1:** Positioning of representative prior works against key capability dimensions.

Work	Slot-Level Adaptive Rate	Channel-Data Decoupling	ISAC Context in Encoder
Xie and Qin [2]	×	×	×
Bourtsoulatze et al. [5]	×	×	×
Choi et al. [12]	✓(Variable rate)	×	×
FedProx [6]	×	×	×
Oort [3]	×	×	×
Su et al. [13]	✓	×	×
Wang et al. [10]	✓	×	×
**This work**	✓	✓	✓

**Table 2:** Computational overhead per sample.

Component	Location	MACs	Latency
ConvNeXt-V2-Tiny (full forward)	Client	$4.5\times 10^{9}$	∼3.5 ms
FiLM MLP (6→256→1536)	Client	$3.95\times 10^{5}$	∼0.03 ms
Element-wise modulation (L×d)	Client	$3.76\times 10^{4}$	<0.01 ms
Token scoring + Gumbel-TopK	Client	$3.79\times 10^{4}$	<0.01 ms
Token projection (768→8)	Client	$1.72\times 10^{5}$	∼0.02 ms
Total SGAM overhead	Client	$6.4\times 10^{5}$	∼0.06 ms
CDFL scheduling (per round, U=20)	Server	$<5\times 10^{3}$	<0.1 ms

**Table 3:** Performance comparison on NuScenes (60 rounds).

Method	Acc	Macro-F1	Worst-F1	*σ*_last-10_
FedAvg	61.4%	0.520	0.215	3.2%
FedProx	63.5%	0.571	0.280	2.8%
Oort-V2X	65.2%	0.623	0.255	2.6%
Proposed	68.9%	0.710	0.465	0.8%

**Table 4:** Ablation analysis of the proposed framework’s core components.

Variant	Acc	M-F1	W-F1	σ
w/o SGAM	62.1%	0.545	0.185	4.1%
w/o hard budget	64.0%	0.590	0.220	3.5%
w/o FiLM	65.8%	0.635	0.310	2.2%
w/o CDFL	65.3%	0.615	0.275	2.8%
w/o IPW	66.5%	0.655	0.340	1.9%
w/o sens loss	67.8%	0.690	0.410	1.2%
Full	68.9%	0.710	0.465	0.8%

**Table 5:** Selection frequency by quadrant (% per round).

Method	$Q_{1}$	$Q_{2}$	$Q_{3}$	$Q_{4}$
FedAvg	15%	25%	28%	32%
Oort-V2X	8%	18%	32%	42%
Proposed	22%	24%	27%	27%

**Table 6:** CDFL scalability and approximation performance.

Fleet Size *U*	Exact Latency	Approx. Latency	Speedup	Objective Retained
20	0.1 ms	0.1 ms	1.0×	100.0%
100	0.6 ms	0.3 ms	2.0×	99.2%
1000	45.2 ms	4.2 ms	10.7×	98.5%
5000	1156.4 ms	21.6 ms	53.5×	97.1%

**Table 7:** Impact of CSI estimation error ($N_{\mathrm{sc}}=128$).

CSI Error $\sigma_{e}^{2}$	Approx. NMSE	Macro-F1	Worst-F1	Semantic Violation	*σ*_last-10_
0 (perfect)	0%	0.710	0.465	0%	0.8%
0.01	∼1%	0.707	0.458	0%	0.8%
0.05	∼5%	0.699	0.441	0%	1.0%
0.10	∼10%	0.689	0.420	0%	1.3%

**Table 8:** Sensitivity to the number of OFDM subcarriers. The upper $K_{\max}$ of 49 for $N_{\mathrm{sc}}=256$ reflects the architectural ceiling $k_{\mathrm{cap}}=L=49$ rather than the channel capacity limit.

$N_{\mathrm{sc}}$	Observed $K_{\max}$ Range	Mean $K_{u}$	Violation Rate	Macro-F1	Worst-F1
64	[4,24]	13.6	0%	0.676	0.395
128	[8,49]	27.8	0%	0.710	0.465
256	[16,49]	36.4	0%	0.719	0.480

## Data Availability

The NuScenes v1.0-trainval dataset used in this study is publicly available at https://www.nuscenes.org/nuscenes (accessed on 20 March 2026) upon free registration. The data preparation and channel simulation scripts will be released as open-source code upon acceptance of the manuscript.

## Abbreviations

   The following abbreviations are used in this manuscript:

AMC	Adaptive Modulation and Coding
AWGN	Additive White Gaussian Noise
BEV	Bird’s-Eye View
CAV	Connected and Automated Vehicle
CDFL	Channel-Decoupled Federated Learning
CRB	Cramér–Rao Bound
CSI	Channel State Information
EMD	Earth Mover’s Distance
FiLM	Feature-wise Linear Modulation
FIM	Fisher Information Matrix
FL	Federated Learning
IPW	Inverse-Propensity Weighting
ISAC	Integrated Sensing and Communication
JSCC	Joint Source-Channel Coding
LSH	Locality-Sensitive Hashing
MLP	Multilayer Perceptron
NLOS	Non-Line-of-Sight
NMSE	Normalized Mean Square Error
OFDM	Orthogonal Frequency-Division Multiplexing
QoS	Quality of Service
RBF	Radial Basis Function
RSU	Roadside Unit
SDR	Software-Defined Radio
SGAM	Sensing-Guided Adaptive Modulation
SNR	Signal-to-Noise Ratio
STE	Straight-Through Estimator
UAV	Unmanned Aerial Vehicle
V2X	Vehicle-to-Everything

## Appendix A. Algorithm Pseudocode

Algorithm A1 summarizes the complete training procedure covering server-side CDFL scheduling, client-side SGAM-enabled local training, and weighted model aggregation.

**Algorithm A1** Training Procedure
**Require:** Global model $\theta^{\left(0\right)}$, *T* rounds, *U* clients, *K* selected per round 1: **for** t=1 to *T* **do** 2: *// Server: CDFL client scheduling* 3: Collect ${\left\{c_{i}\right\}}_{i=1}^{U}$ from all clients 4: Compute per-round medians of SNR, κ, loss, entropy 5: Partition clients into $Q_{1},\ldots ,Q_{4}$ via Equations (15) and (16) 6: **for** each quadrant j∈{1,2,3,4} **do** 7: $A_{j}\leftarrow$ GreedyFacilityLocation $\left(Q_{j},N_{j}\right)$ 8: **end for** 9: $A\leftarrow {⋃}_{j}A_{j}$; compute weights $\left\{w_{u}\right\}$ via Equation (19) 10: Broadcast $\theta^{\left(t\right)}$ to clients in A 11: *// Client: local training with SGAM* 12: **for** each u∈A in parallel **do** 13: $\theta_{u}\leftarrow \theta^{\left(t\right)}$ 14: **for** epoch =1 to *E* **do** 15: **for** each batch $\left(x,y\right)\in D_{u}$ **do** 16: $Z_{\mathrm{base}}\leftarrow \mathrm{Backbone}\left(x\right)$ 17: Construct $q_{u}$ via Equation (9) 18: $\left[\gamma ,\beta \right]\leftarrow {\mathrm{MLP}}_{\mathrm{FiLM}}\left(q_{u}\right)$ 19: $\tilde{Z}\leftarrow \gamma ⊙Z_{\mathrm{base}}+\beta$ 20: $K_{\max}\leftarrow$ Equation (7) from current $|S_{u}^{\left(t\right)}|$ 21: $K_{u}\leftarrow \mathrm{clip}\left(K_{\max}+\Delta K\left(q_{u}\right),\;K_{\min},\;K_{\max}\right)$ 22: $m\leftarrow \mathrm{GumbelTopK}(\mathrm{scores},K_{u})$ 23: $\alpha \leftarrow \mathrm{softmax}(\mathrm{gate}\left(\tilde{Z}\right)⊙m)$ 24: $z\leftarrow \sum_{l}\alpha_{l}{\tilde{z}}_{l}$ 25: Compute L via Equation (25) 26: $\theta_{u}\leftarrow \theta_{u}-\eta \nabla_{\theta_{u}}L$ 27: **end for** 28: **end for** 29: Upload $\theta_{u}$ to server 30: **end for** 31: *// Server: weighted aggregation* 32: $\theta^{\left(t+1\right)}\leftarrow$ Equation (20) 33: **end for** 34: **return** $\theta^{\left(T\right)}$

## Appendix B. Symbol Reference

Table A1 lists the principal symbols that appear in the formulation.

**Table A1:** Key symbols and descriptions.

Symbol	Description
$S_{u}^{\left(t\right)}$	Subcarrier set allocated to client *u* at round *t*
$h_{u,k}^{\left(t\right)}$	Channel coefficient for client *u*, subcarrier *k*, round *t*
$J_{u}$	Fisher information matrix for client *u*
$\mathrm{CRB}(p_{u})$	Cramér–Rao bound for sensing parameter vector $p_{u}$
$\xi_{u}$	Sensing confidence score
$B_{\mathrm{slot}}$	Usable bits per OFDM slot
$b_{\mathrm{token}}$	Bits per semantic token (=$d_{b}\times q$)
$K_{\max}^{\left(t\right)}$	Instantaneous token budget from channel allocation
$q_{u}$	Six-dimensional condition vector
γ,β	FiLM scale and shift parameters
m	Binary token selection mask
$K_{u}$	Number of tokens selected for client *u*
ΔK	Learnable budget adjustment
${\mathrm{ChQ}}_{i}$, ${\mathrm{SemD}}_{i}$	Channel quality/data complexity index
$Q_{j}$, $N_{j}$	Quadrant *j*; selection quota for quadrant *j*
$w_{i}$	Inverse-propensity weight for client *i*
$L_{\mathrm{comm}}$	Combined task loss
$L_{\mathrm{sens}}$	Sensing regularizer loss
$L_{\mathrm{UW}}$	Uncertainty-weighted combined loss
$\sigma_{c},\sigma_{s}$	Learnable uncertainty parameters
$\Gamma^{2}$	Client heterogeneity bound
$\delta_{\mathrm{sub}}$	Submodular approximation ratio (=1−1/e)
